# Incidence and mortality trends of leukemia and lymphoma in Croatia, 1988-2009

**DOI:** 10.3325/cmj.2012.53.115

**Published:** 2012-04

**Authors:** Ina Novak, Ozren Jakšić, Tomislav Kuliš, Katarina Batinjan, Ariana Znaor

**Affiliations:** 1 Andrija Štampar School of Public Health, University of Zagreb School of Medicine, Zagreb, Croatia; 2 Department of Hematology, Dubrava University Hospital, Zagreb, Croatia; 3 Department of Urology, University Hospital Centre Zagreb, University of Zagreb School of Medicine, Zagreb, Croatia; 4 Croatian National Cancer Registry, Croatian National Institute of Public Health, Zagreb, Croatia

## Abstract

**Aim:**

To investigate the time trends of leukemia and lymphoma in Croatia from 1988-2009, compare them with trends in other populations, and identify possible changes.

**Methods:**

The data sources were the Croatian National Cancer Registry for incidence data, Croatian Bureau of Statistics for the numbers of deaths, and United Nations population estimates. Joinpoint regression analysis using the age-standardized rates was used to analyze incidence and mortality trends.

**Results:**

Acute lymphoblastic leukemia and chronic lymphocytic leukemia incidence did not significantly change. Acute myeloid leukemia incidence significantly increased in women, with estimated annual percentage change (EAPC) of 2.6% during the whole period, and in men since 1998, with EAPC of 3.2%. Chronic myeloid leukemia incidence significantly decreased in women (EAPC -3.7%) and remained stable in men. Mortality rates were stable for both lymphoid and myeloid leukemia in both sexes. Hodgkin lymphoma non-significantly increased in incidence and significantly decreased in mortality (EAPCs of -5.6% in men and -3.7% in women). Non-Hodgkin lymphoma significantly increased in incidence in women (EAPC 3.2%) and non-significantly in men and in mortality in both men (EAPC 1.6%) and women (EAPC 1.8%).

**Conclusion:**

While Croatia had similar leukemia and lymphoma incidence trends as the other countries, the mortality trends were less favorable than in Western Europe. The lack of declines of leukemia incidence and non-Hodgkin lymphoma mortality could be attributed to late introduction of optimal therapies. As currently the most up-to-date diagnostics and treatments are available and covered by health insurance, we expect more favorable trends in the future.

Leukemias and lymphomas contribute 5% to the overall cancer incidence in Croatia (1). They comprise disease entities diverse in etiology, incidence, prognosis, and treatment. The four major leukemia subtypes include acute lymphoblastic leukemia (ALL), chronic lymphocytic leukemia (CLL), acute myeloid leukemia (AML), and chronic myeloid leukemia (CML), while lymphomas include Hodgkin lymphoma (HL) and non-Hodgkin lymphoma (NHL).

Estimated 5-year relative survival for patients diagnosed between 2000 and 2002 in Europe, according to EUROCARE-4 results, is 43.4% for the overall group of leukemias. CLL has the highest 5-year survival rate (70.2%), followed by CML (37.2%), ALL (28.8%), and AML (15.8%). Five-year survival rates for lymphomas were 81.9% for HL and 53.6% for NHL (2).

Recognized environmental risk factors for leukemia are exposure to ionising radiation (3-5), chemicals such as benzene (6), pesticides (7), chemotherapy (8), cigarette smoking (9), genetic disorders (10,11), family history in case of CLL (12), infection with HTLV-I (13), socio-economic status (14), and obesity (15). However, those risk factors could explain only a minority of cases, and leukemia etiology remains largely unknown. Environmental risk factors for NHL are exposure to pesticides, solvents (16,17) and HIV infection (18), while those for HL include HIV (19) and Epstein-Barr virus infection (20).

The last decades brought significant improvements in diagnosis and treatment of leukemias and lymphomas. The aim of our study was to investigate the time trends of leukemia and lymphoma in Croatia from 1988-2009, compare them with trends in other populations, and identify possible changes.

## Materials and methods

### Data sources

Incidence data for the period 1988-2009 were obtained from the Croatian National Cancer Registry. The Registry, founded in 1959, covers the whole Croatian population (approximately 4.4 million persons), and relies on mandatory cancer notifications from primary and secondary health care sources and death certificates from the Croatian Bureau of Statistics. The Registry contributed data to the last three volumes of the Cancer Incidence in Five Continents series (21-23). Leukemia and lymphoma were defined as ICD-9 codes 201 (HL), 202 (NHL), 204 (lymphoid leukemia), 204.0 (ALL), 204.1 (CLL), 205 (myeloid leukemia), 205.0 (AML), 205.1 (CML) and ICD-10 codes C81 (HL), C82-85 (NHL), C91 (lymphoid leukemia), C91.0 (ALL), C91.1 (CLL), C92 (myeloid leukemia), C92.0 (AML), C92.1 (CML) (24). The numbers of cancer deaths were obtained from WHO mortality database, and were not available with 4 ICD digits (25). For calculating age-specific rates we used the United Nations population estimates (26).

### Statistical analysis

Age-standardized rates of cancer incidence in Croatia were calculated by the direct standardization method, using the world standard population as a reference (27). To describe incidence and mortality trends by calendar period, we carried out joinpoint regression analysis using the Joinpoint Regression Software (28). The analysis included logarithmic transformation of the rates, standard error, maximum number of five joinpoints, and minimum of four years between two joinpoints. All other program parameters were set to default values. The aim of the approach is to identify possible joinpoints, where a significant change in the trend occurs. The method identifies joinpoints based on regression models with 0-5 joinpoints. The final model selected was the most parsimonious of these, with the estimated annual percent change (EAPC) based on the trend within each segment (12). To quantify the trend over the whole period, the average annual percent change (AAPC) was calculated. The AAPC is computed as a geometric weighted average of the EAPC trend analysis, with the weights equal to the lengths of each segment during the prespecified fixed interval. If an AAPC lies entirely within a single joinpoint segment, the AAPC is equal to the EAPC for that segment. In these cases, we chose to report the EAPC (29). In describing trends, the terms “significant increase” or “significant decrease” signify that the slope of the trend was statistically significant (*P* < 0.05). For non-statistically significant trends (*P* > 0.05), we used the terms “stable” (for EAPC between -0.5% and 0.5%), “non-statistically significant increase” (for EAPC>0.5%), and “non-statistically significant decrease” (for EAPC<-0.5%). All statistical tests were two sided.

## Results

Between 1988 and 2009, there were 785 cases of ALL in men and 612 in women, 1881 cases of CLL in men and 1494 in women, 1084 cases of AML in men and 1057 in women, 612 cases of CML in men and 510 in women, with a total of 8035 cases, 4362 male and 3673 female. The most common leukemia type was CLL, comprising 42% of leukemias, followed by AML with 27%, ALL with 17%, and CML with 14%.

The age-standardized incidence rates for all subtypes were higher in men than in women, more pronounced for chronic leukemias (Table 1). Incidence rates increased with age, and majority of newly diagnosed cases in the period 2005-2009 were in the 65+ age group, except for ALL, which presented a bimodal pattern with 51% cases aged 0-19 and 21% cases in the 65+ age group. For CLL, 75% patients were older than 65 years (data not shown).

**Table 1:** Average annual numbers of new cases and age-standardized rates (ASR) per 100 000 of leukemia and lymphoma in Croatia, 1988-1992 and 2005-2009 (using world standard population)

	Men					Women				
	1988-1992		2005-2009			1988-1992		2005-2009		
	N	ASR	N	ASR	change (%)	N	ASR	N	ASR	change (%)
ALL	37	1.99	36	2.25	13	26	1.37	28	1.64	20
CLL	78	2.75	98	2.59	-6	64	1.50	72	1.20	-20
AML	34	1.41	75	2.30	63	36	1.06	64	1.63	54
CML	23	0.85	25	0.74	-13	27	0.77	17	0.37	-52
HL	43	1.77	57	2.32	31	36	1.45	64	2.56	77
NHL	132	5.11	215	6.96	36	120	3.53	234	5.57	58

*Abbreviations: ALL – acute lymphoblastic leukemia; CLL – chronic lymphocytic leukemia; AML – acute myeloid leukemia; CML – chronic myeloid leukemia; HL – Hodgkin lymphoma; NHL – non-Hodgkin lymphoma.

**Figure 1:**
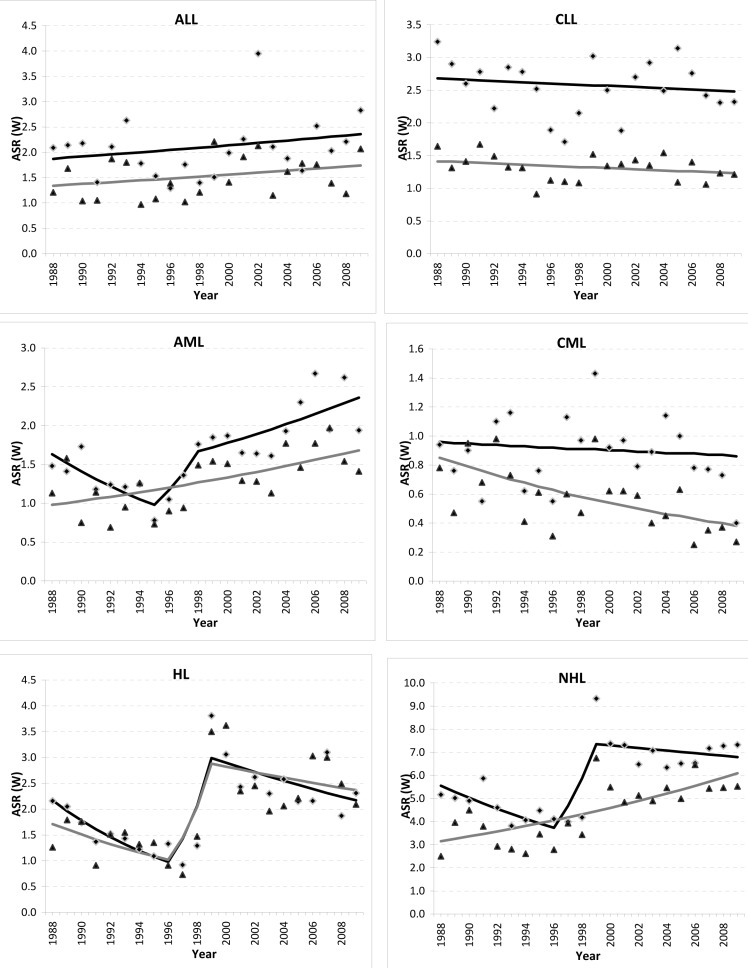
Incidence trends of hematopoietic malignancies in Croatia, 1988-2009, rhombs – male; triangles – female. ASR (W) – age-standardized rate per 100 000 (using world standard population).

ALL incidence showed a slight non-significant increase, while CLL incidence showed decreases in both sexes (Table 2, Figure 1). Between the first and the last observed five-year period, AML incidence rate increased by 63% in men and 54% in women (Table 1). While the female AML incidence was steadily increasing, with EAPC of 2.6% (95% confidence interval [CI], 1.0 to 4.3), in men three different trend segments were observed, with a significant increase of 3.2% (95% CI, 0.3 to 6.2) annually observed since 1998. CML incidence in women decreased by 52% between the first and the last five-year period, with a continuous significant decrease of 3.7% (95% CI, -5.9 to -1.4) annually, while it remained stable in men (Table 2, Figure 1).

**Table 2:** Average annual percentage changes (AAPC) of incidence and mortality of leukemia and lymphoma in Croatia with 95% confidence intervals (CI), 1988-2009

	Incidence		Mortality		
	AAPC	95% CI		AAPC	95% CI
ALL:			ALL+CLL		
male	1.1	-0.7 to 3.0		-0.3	-1.4 to 0.8
female	1.3	-0.6 to 3.1		0.1	-1.0 to 1.2
CLL:					
male	-0.4	-1.5 to 0.8			
female	-0.7	-1.7 to 0.4			
AML:			AML+CML		
male	1.8	-6.1 to 10.3		0.2	-0.8 to 1.2
female	2.6^†^	1.0 to 4.3		0.3	-0.8 to 1.3
CML:					
male	- 0.5	-2.6 to 1.6			
female	- 3.7^†^	- 5.9 to -1.4			
HL:					
male	0.0	-7.2 to 7.8		-5.6^†^	-7.3 to -3.9
female	1.5	-9.9 to 14.4		-3.7^†^	-6.3 to -1.0
NHL:					
male	1.0	-3.0 to 5.1		1.6^†^	0.8 to 2.4
female	3.2^†^	1.7 to 4.7		1.8^†^	0.8 to 2.9

*Abbreviations: ALL – acute lymphoblastic leukemia; CLL – chronic lymphocytic leukemia; AML – acute myeloid leukemia; CML – chronic myeloid leukemia; HL – Hodgkin lymphoma; NHL – non-Hodgkin lymphoma.

†Significant result.

**Figure 2:**
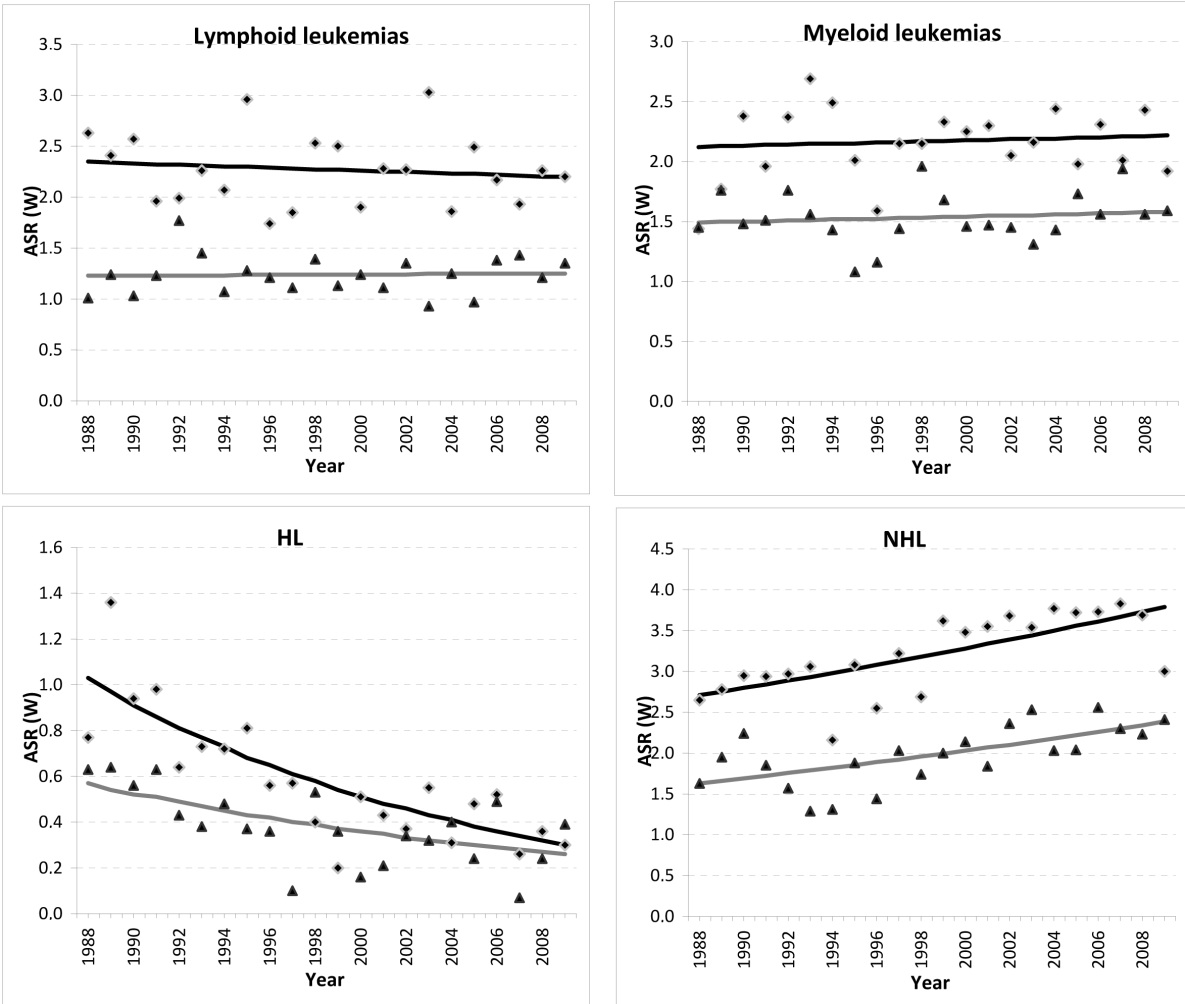
Mortality trends of hematopoietic malignancies in Croatia, 1988-2009, rhombs – male; triangles – female. ASR (W) – age-standardized rate per 100 000 (using world standard population).

A total of 5691 deaths were identified, 2759 from lymphoid leukemias (1524 men and 1235 women) and 2932 from myeloid leukemias (1507 men and 1425 women). No significant changes of mortality trends for either lymphatic or myeloid leukemias were observed in either sex (Table 2, Figure 2).

NHL was the most common hematopoietic malignancy analyzed, with 3791 diagnosed men and 3746 women. For both NHL and HL, ASRs were higher for men. NHL incidence increased with age, while HL showed a bimodal pattern, with the first peak in the age group 20-29 years, and the second peak in the age group 60-69 years (data not shown). Between the first and the last five-year period, the overall increase in NHL incidence was 26% in men and 24% in women (Table 1). For men, three different trend segments were identified, with AAPC of 1% (95% CI, -3 to 5.1), while in women the incidence increased significantly by 3.2% (95% CI, 1.7 to 4.7) without sharp changes of trend. For HL in both sexes, a non-significant increase in the incidence with three different trends was also observed. While NHL mortality steadily increased in both sexes, with EAPC of 1.6% (95% CI, 0.8 to 2.4) in men and 1.8% (95% CI, 0.8 to 2.9) in women, HL mortality markedly decreased, with EAPC of -5.6% (95% CI, -7.3 to -3.9) in men and -3.7% (95% CI, -6.3 to -1.0) in women (Table 2, Figure 1). The overall decreases in HL mortality between the first and the last five-year periods were more than 50% in both sexes (Table 3).

**Table 3:** Average annual numbers of deaths and age-standardized rates (ASR) per 100 000 of leukemia and lymphoma in Croatia, 1988-1992 and 2005-2009 (using world standard population)

	Men					Women				
	1988-1992		2005-2009			1988-1992		2005-2009		
	N	ASR	N	ASR	change (%)	N	ASR	N	ASR	change (%)
Lymphoid leukemia	59	2.31	80	2.21	-4	49	1.26	68	1.27	1
Myeloid leukemia	52	1.98	76	2.13	8	57	1.59	76	1.68	6
HL	25	0.94	13	0.38	-60	20	0.58	10	0.29	-50
NHL	76	2.86	128	3.59	26	70	1.85	121	2.31	25

*Abbreviations: HL – Hodgkin lymphoma, NHL – non-Hodgkin lymphoma.

## Discussion

According to GLOBOCAN estimates for the year 2008, age-standardized incidence rate of leukemia in Croatia is 8.6/100 000 in men and 5.3/100 000 in women, which is slightly higher than the European average for men (8.3/100 000) and lower than the European average for women (5.6/100 000). The lowest incidence rate in Europe in both sexes is found in Central and Eastern Europe. Age-standardized mortality rate in Croatian men is 5.3/100 000, compared to the European average of 4.9/100 000, while the rate in women of 3.0/100 000 equals the European average. The lowest rates in both sexes are observed in Northern Europe (30). HAEMACARE project group, using five large categories of myeloid and five categories of lymphoid malignancies in the period 2000-2002 observed the lowest leukemia rates in Eastern Europe, which they assumed to be caused by underreporting (31).

We found higher age-standardized incidence and mortality rates in men than in women. The age and sex distribution patterns of leukemia in Croatia correspond to those observed in other countries (32,33).

Incidence trends of leukemia types vary across countries. The stable ALL incidence in Croatia corresponds to trends reported in other countries, like the US, UK, and the Netherlands (32,34,35). Increasing CLL incidence rates were reported in Denmark (33) and in Dutch women (35). The latter has been attributed to increased surveillance in the scope of the mammography screening program (35). AML incidence in Croatia increased in both sexes, which is consistent with a Danish study covering the period from 1943 to 2003 (33). It was also increasing in the US (36) from the mid-1980s to the early 2000. However, in the UK it was declining for from 1984 to 1993 (34), while in French Department Cote d´Or it showed a relatively stable incidence from 1980 to 2004 (37). CML incidence trends show lower variation between countries, ie, several studies showed continued declines, similar to our findings (32-34).

Given the variety of risk factors implicated in the etiology of leukemias and lymphomas, it is hard to hypothesize possible reasons for the observed incidence trends. In addition to true changes of incidence, they might be caused by changes of diagnostic procedures or increased surveillance due to screening programs, as well as changes of classification or reassignment of one diagnostic entity to the other (31,35,38). Based on the results from 13 European countries, the marked increase in NHL incidence trends could be partly explained by reassignment of HL as NHL and consequent decreases in HL trends (38). The increase in NHL incidence in Croatia was not as prominent as reported in other countries; however, while the incidence has leveled off in the US and most of the European countries, in Croatia it still continues (36,38). HL incidence trends vary across countries, and are usually less stable due to smaller number of cases (38). Three different trend segments observed in male NHL and HL incidence in both sexes in Croatia probably represent a consequence of registration, rather than changes in background incidence rates. Underreporting of lymphomas was evident during the war in Croatia (1991-1995), while the peak of incidence in 1999 can be attributed to the introduction of a new data source (1), and possible registration of some prevalent cases. We cannot assess the extent of possible reassignment of HL to NHL diagnosis; however, the divergent NHL and HL mortality trends observed in Croatia would support this hypothesis.

Mortality trends, in addition to changes in incidence or classification of disease, reflect improvements of treatment and survival. Decreasing leukemia mortality trends were reported in Western countries between late 1960s and late 1990s (39,40). In the same period, in Eastern European countries, the decline was limited to younger age-groups (15-44 years), while the age-standardized rates did not significantly change. When we analyzed age-specific mortality trends of our study population, no significant decreases were observed even in the youngest age-group (15-44) for either sex or leukemia type.

Decreases in mortality are preceded by improvements of survival. The recent increasing trends of leukemia survival in the US and Europe have been attributed to improvements in care, more specific diagnosis and treatment, as well as improved supportive care (41-43). Even though the improvements of leukemia survival are more prominent in younger patients, in the study of recent survival trends in younger patients in Croatia, no improvements have been observed in the period between 2000 and 2006 (44).

While the decreasing mortality trends for HL have been reported throughout Europe (45), the declines in NHL mortality in the European Union have started only from the mid-1990s. In Eastern European countries, as well as in Croatia, increasing mortality trends are still observed (46).

The last decades have witnessed several major breakthroughs in the treatment of leukemias and lymphomas, such as introduction of allogenic hematopoietic stem cell transplantation (usually restricted to patients younger than 65 years), and more recent introduction of tyrosine-kinase inhibitor imatinib in the treatment of CML in 2001 (47), and monoclonal antibodies (rituximab) in the treatment of NHL in 1997 (48). After the launch in the US, these treatments were introduced to Europe (49). In Croatia, imatinib was introduced in the first line of treatment only in 2003, and rituximab in 2006. Also the bone marrow transplantation rates in Croatia were very low. In 2003, the hematopoietic allogenic stem cell transplant rate in Croatia was 56 per 10 million, which is about two- to 3-fold lower than in Western European countries (50). The transplantation rates increased only after the “I want life!” campaign was started in 2006, during which the number of voluntary bone marrow donors in Croatia increased from 150 to 20 000 (51), and with an increased number of centers for stem cell transplantation (52). However, these recent improvements have not reflected on our data.

The limitation of our study was the inability to distinguish between the mortality of acute and chronic leukemias, and detect possible improvements in a specific leukemia type, such as in the SEER9 database, where divergent trends of mortality from myeloid leukemias are observed (increasing AML mortality and markedly decreasing CML mortality) (36).

In spite of the long tradition and high expertise of Croatian hematology (53), the delayed introduction of optimal treatment modalities resulted in less favorable mortality trends than in Western European countries. As currently the most up-to date diagnostics and best tailored treatments are available and covered by health insurance, we hope that, in addition to already declining HL mortality trend, improvements of leukemia and NHL survival and subsequent declines of mortality will follow.

## References

[1] Croatian National Cancer Registry, Croatian National Institute of Public Health. Cancer incidence in Croatia 1975-2009. Bulletins No 1-34. Zagreb, Croatia: Croatian National Institute of Public Health; 1985-2010.

[2] Brenner H, Francisci S, de Angelis R, Marcos-Gragera R, Verdecchia A, Gatta G (2009). Long-term survival expectations of cancer patients in Europe in 2000-2002.. Eur J Cancer.

[3] Preston DL, Kusumi S, Tomonaga M, Izumi S, Ron E, Kuramoto A (1994). Cancer incidence in atomic bomb survivors. Part III. Leukemia, lymphoma and multiple myeloma, 1950-1987.. Radiat Res.

[4] Fabrikant JI (1991). The carcinogenic risks of low-LET and high-LET ionizing radiations.. J Radiat Res (Tokyo).

[5] Daniels RD, Schubauer-Berigan MK (2011). A meta-analysis of leukemia risk from protracted exposure to low-dose gamma radiation.. Occup Environ Med.

[6] Khalade A, Jaakkola MS, Pukkala E, Jaakkola JJ (2010). Exposure to benzene at work and the risk of leukemia: a systematic review and meta-analysis. Environ Health.

[7] Turner MC, Wigle DT, Krewski D (2011). Residential pesticides and childhood leukemia: a systematic review and meta-analysis.. Cien Saude Colet.

[8] Curtis RE, Boice JD, Stovall M, Bernstein L, Greenberg RS, Flannery JT (1992). Risk of leukemia after chemotherapy and radiation treatment for breast cancer.. N Engl J Med.

[9] Kane EV, Roman E, Cartwright R, Parker J, Morgan G (1999). Tobacco and the risk of acute leukemia in adults.. Br J Cancer.

[10] Deschler B, Lübbert M (2006). Acute myeloid leukemia: epidemiology and etiology.. Cancer.

[11] Xavier AC, Taub JW (2010). Acute leukemia in children with Down syndrome.. Haematologica.

[12] Crowther-Swanepoel D, Houlston RS (2010). Genetic variation and risk of chronic lymphocytic leukemia.. Semin Cancer Biol.

[13] Takatsuki K (1995). Adult T-cell leukemia.. Intern Med.

[14] Poole C, Greenland S, Luetters C, Kelsey JL, Mezei G (2006). Socioeconomic status and childhood leukemia: a review.. Int J Epidemiol.

[15] Lichtman MA (2010). Obesity and the risk for a haematologicallogical malignancy: leukemia, lymphoma, or myeloma.. Oncologist.

[16] Cocco P, t'Mannetje A, Fadda D, Melis M, Becker N, de Sanjose S (2010). Occupational exposure to solvents and risk of lymphoma subtypes: results from the Epilymph case-control study.. Occup Environ Med.

[17] Besson H, Brennan P, Becker N, De Sanjose S, Nieters A, Font R (2006). Tobacco smoking, alcohol drinking and Hodgkin's lymphoma: a European multi-centre case-control study (EPILYMPH).. Br J Cancer.

[18] Cote TR, Biggar RJ, Rosenberg PS, Devesa SS, Percy C, Yellin FJ (1997). Non-Hodgkin's lymphoma among people with AIDS: incidence, presentation and public health burden. AIDS/Cancer Study Group.. Int J Cancer.

[19] International Agency for Research on Cancer. Monographs on the evaluation of carcinogenic risk to humans, Vol 67. Human immunodeficiency virus and human lymphotrophic T-cell viruses. Lyon (France): IARC press; 1996.

[20] International Agency for Research on Cancer. Monographs on the evaluation of carcinogenic risk to humans, Vol 70. Epsterin-Barr virus and Kaposi’s sarcoma Herpesvirus/Human Herpesvirus 8. Lyon (France): IARC press; 1998.

[21] Curado MP, Edwards B, Shin HR, Storm H, Ferlay J, Heanue M, et al, editors. Cancer incidence in five continents, Vol IX (IARC Scientific Publications No. 160). Lyon (France): IARC; 2007.

[22] Parkin DM, Whelan SL, Ferlay J, Raymond L, Young J, editors. Cancer incidence in five continents, Vol VII (IARC Scientific Publications No. 143). Lyon (France): IARC; 1997.

[23] Parkin DM, Whelan SL, Ferlay J, Teppo L, Thomas DB, editors. Cancer Incidence in five continents, Vol VIII (IARC Scientific Publications No. 155). Lyon (France): IARC; 2002.

[24] International Classification of Diseases (ICD). Available from: http://www.who.int/classifications/icd/en/ Accessed: February 12, 2011.

[25] World Health Organization. World Health Organization mortality database. WHO Statistical Information System. 2011. Available from: http://www.who.int/whosis/mort/download/en/index.html*.* Accessed: April 12, 2012.

[26] United Nations. World population prospects, the 2010 revision. United Nations Population Division Department of Economic and Social Affairs. Available from: http://www.un.org/esa/population/unpop.htm Accessed: February 12, 2011.

[27] Doll R, Payne P, Waterhouse J. Cancer incidence in five continents: a technical report. New York (NY): Springer, 1966.

[28] National Cancer Institute. Joinpoint regression program. 3.4.2 ed. Bethesda: Statistical Research and Applications Branch, National Cancer Institute, 2009.

[29] Kim HJ, Fay MP, Feuer EJ, Midthune DN (2000). Permutation tests for joinpoint regression with applications to cancer rates.. Stat Med.

[30] Ferlay J, Shin HR, Bray F, Forman D, Mathers C, Parkin DM. GLOBOCAN 2008, Cancer Incidence and Mortality Worldwide: IARC CancerBase No. 10. In*:* 2010. Lyon, France: International Agency for Research on Cancer; 2010. Available from: http://globocan.iarc.fr*.* Accessed: February 12, 2011.

[31] Sant M, Allemani C, Tereanu C, De Angelis R, Capocaccia R, Visser O, HAEMACARE Working Group (2010). Incidence of haematologic malignancies in Europe by morphologic subtype: results of the HAEMACARE project.. Blood.

[32] Xie Y, Davies SM, Xiang Y, Robison LL, Ross JA (2003). Trends in leukemia incidence and survival in the United States (1973-1998).. Cancer.

[33] Thygesen LC, Nielsen OJ, Johansen C (2009). Trends in adult leukemia incidence and survival in Denmark, 1943-2003.. Cancer Causes Control.

[34] McNally RJ, Roman E, Cartwright RA (1999). Leukemias and lymphomas: time trends in the UK, 1984-93.. Cancer Causes Control.

[35] van den Broek EC, Kater AP, van de Schans SA, Karim-Kos HE, Janssen-Heijnen ML, Peters WG (2012). Chronic lymphocytic leukemia in the Netherlands: Trends in incidence, treatment and survival, 1989-2008.. Eur J Cancer.

[36] Surveillance E, Results E. (SEER) Program SEER*Stat Database: Mortality – All COD, Aggregated With State, Total U.S. (1969-2008) Katrina/Rita Population Adjustment, National Cancer Institute, DCCPS, Surveillance Research Program, Cancer Statistics Branch, released October 2011. Available from: http://www.seer.cancer.gov Accessed: December 2, 2011.

[37] Maynadie M, Girodon F, Manivet-Janoray I, Mounier M, Mugneret F, Bailly F (2011). Twenty-five years of epidemiological recording on myeloid malignancies: data from the specialized registry of hematologic malignancies of Cote d'Or (Burgundy, France).. Haematologica.

[38] Adamson P, Bray F, Costantini AS, Tao MH, Weiderpass E, Roman E (2007). Time trends in the registration of Hodgkin and non-Hodgkin lymphomas in Europe.. Eur J Cancer.

[39] Imamura Y, Mizuno S (2005). Comparison of leukemia mortality in five countries: France, Italy, Japan, UK and USA from the WHO Mortality Database (1960-2000).. Jpn J Clin Oncol.

[40] Levi F, Lucchini F, Negri E, Barbui T, La Vecchia C (2000). Trends in mortality from leukemia in subsequent age groups.. Leukemia.

[41] Pulte D, Gondos A, Brenner H (2008). Improvements in survival of adults diagnosed with acute myeloblastic leukemia in the early 21st century.. Haematologica.

[42] Brenner H, Gondos A, Pulte D (2008). Recent trends in long-term survival of patients with chronic myelocytic leukemia: disclosing the impact of advances in therapy on the population level.. Haematologica.

[43] Brenner H, Gondos A, Pulte D (2008). Trends in long-term survival of patients with chronic lymphocytic leukemia from the 1980s to the early 21st century.. Blood.

[44] Znaor A, Brenner H, Holleczek B, Gondos A (2012). Has there been progress in cancer care in Croatia? Assessing outcomes in a partially complete mortality follow-up setting.. Eur J Cancer.

[45] Bosetti C, Levi F, Ferlay J, Lucchini F, Negri E, La Vecchia C (2009). The recent decline in mortality from Hodgkin lymphomas in central and eastern Europe.. Ann Oncol.

[46] Bosetti C, Levi F, Ferlay J, Lucchini F, Negri E, La Vecchia C (2008). Incidence and mortality from non-Hodgkin lymphoma in Europe: the end of an epidemic?. Int J Cancer.

[47] Cohen MH, Williams G, Johnson JR, Duan J, Gobburu J, Rahman A (2002). Approval summary for imatinib mesylate capsules in the treatment of chronic myelogenous leukemia.. Clin Cancer Res.

[48] Forstpointner R, Dreyling M, Repp R, Hermann S, Hänel A, Metzner B (2004). The addition of rituximab to a combination of fludarabine, cyclophosphamide, mitoxantrone (FCM) significantly increases the response rate and prolongs survival as compared with FCM alone in patients with relapsed and refractory follicular and mantle cell lymphomas: results of a prospective randomized study of the German Low-Grade Lymphoma Study Group.. Blood.

[49] Jonsson B, Wilking N (2007). A global comparison regarding patient access to cancer drugs.. Ann Oncol.

[50] Gratwohl A, Baldomero H, Labar B, Apperley J (2004). Urbano-Ispizua A for the Accreditation Committee of the European Group for Blood and Marrow Transplantation (EBMT). Evolution of Hematopoietic Stem Cell Transplantation in Eastern andWestern Europe from 1990 to 2003. A Report from the EBMT Activity Survey.. Croat Med J.

[51] Ana Rukavina Foundation. Available from: http://www.zaklada-ana-rukavina.hr/content/en/aktivnosti_zaklade/zelim_zivot.aspx#1 Accessed: April 13, 2012.

[52] Gratwohl A, Baldomero H, Schwendener A, Gartwohl M, Apperley J, Frauendorfer K (2011). The EBMT activity survey 2008 impact of team size, team density and new trends.. Bone Marrow Transplant.

[53] Minigo H (2007). Role of hematology the Croatian health care system. Lijec Vjesn.

